# Imaging carbon nanostructures’ reactivity: a complementary strategy to define chemical structure

**DOI:** 10.1098/rsos.180605

**Published:** 2018-08-08

**Authors:** Verónica Pérez-Luna, Mario Cisneros, Carla Bittencourt, Izcoatl Saucedo-Orozco, Mildred Quintana

**Affiliations:** 1 Instituto de Física, Universidad Autónoma de San Luis Potosí, Manuel Nava 6, Zona Universitaria San Luis Potosí, San Luis Potosí SLP 78290, México; 2 Chemie des Interactions Plasma-Surface, University of Mons, Avenue Nicolas Copernic, 1, 7000 Mons, Belgium; 3 Microscopia de Alta Resolución, Centro de Investigación en Ciencias de la Salud y Biomedicina, Universidad Autónoma de San Luis Potosí, Av. Sierra Leona 550, San Luis Potosí SLP 78210, México

**Keywords:** carbon nanotubes, graphene, graphene oxide gold nanoparticles, chemical functionalization, transmission electron microscopy

## Abstract

In the search for the integration of carbon nanostructures in composite and functional materials, covalent organic reactions are successfully performed. This approach resulted in the construction of tailored chemical interfaces facilitating incorporation of nanocarbons. By a combination of different characterization techniques, such as high-resolution X-ray photo-spectroscopy, thermogravimetric analysis, Raman spectroscopy, UV-vis-nIR, and fluorescence spectroscopies, it is possible to identify and quantify the functional moieties covalently attached to the carbon frame. However, the determination of the structural conformation of functionalized nanostructures remains a difficult task. In this work, we present a straightforward methodology to visualize by transmission electron microscopy the functional moieties covalently attached to the carbon network in carbon nanotubes and graphene. The identification of the functionalities occurs in colloidal dispersions by using gold nanoparticles (AuNPs) as discriminating markers by molecular recognition or by the direct growth of AuNPs on the oxygenated moieties. This methodology, in combination with other characterization analysis, is expected to improve the design of hierarchical interfaces by the spatial localization of the functionalities responsible for colloidal stabilization in solvents with different polarities, different from their homogeneous incorporation into different matrices.

## 1. Introduction

During the last decades the insertion of the fascinating chemical and physical properties of one-dimensional carbon nanotubes (CNTs) and two-dimensional graphene in functional materials has attracted enormous interest. As produced, large intermolecular forces maintain pristine carbon nanostructures forming aggregates, making the incorporation of their remarkable properties in composites challenging. Individual CNT and graphene sheets possess high surface areas, flexibility, electrical and thermal conductivities, properties not observed in bundles of CNTs [1,2] or in graphite nanocrystals [3,4]. To a large extent, the surface properties like hydrophobicity, aromatic stacking and surface topology from the structural to the atomic level direct the performance and functionality of carbon nanostructures in composite materials [5] and in complex interacting systems, i.e. at the nano–bio boundary [6]. In this direction, surface chemical functionalization provides an effective strategy to regulate the interface between carbon nanostructures and other materials, compounds or molecules. Thus, understanding carbon nanostructures’ chemical reactivity is of paramount importance in material design. The main characteristic associated with most carbon nanomaterials is the presence of pentagons and heptagons in a predominantly hexagonal carbon network leading to positive and/or negative curvature [7]. In fullerenes and CNTs, the curvature has been put in relation with an enhanced reactivity; namely, the reactivity increases with increasing degree of curvature [8]. In graphene, however, the edges are usually considered the most reactive sites as only a few defects are observed on the highly crystalline lattice [9]. Then, covalent reactions are presumed to occur preferentially at the graphene edges and CNT tips. The chemical attachment of functional moieties on the reactive sites of graphitic nanostructures is determined by a number of characterization techniques. The quantitative characterization includes thermogravimetric analysis (TGA), X-ray photo-spectroscopy (XPS), Raman, UV-vis-nIR and photoluminescence spectroscopies. However, monitoring the spatial localization of the functional groups covalently attached on the carbon surface has remained a difficult task. In fullerenes, nuclear molecular resonance (NMR) [10] is used with this aim, whereas higher molecular weight and structural conformation limit NMR characterization use in CNTs and graphene.

In the present work, we show the straightforward localization by TEM of carboxylic and amino functional groups covalently grafted to CNTs and graphene by the discriminating attachment of AuNPs on the introduced chemical functional groups. AuNPs are chosen as contrast markers because they are easy to synthesize in different sizes, present high chemical stability and their facile surface functionalization renders them perfect systems for directed self-assembly [11,12]. The identification of the reactive sites is possible due to the selective control of non-covalent interactions at the molecular level or by inducing the growth of AuNPs *in situ* assisted by UV-light irradiation [13]. The localization of the functional groups grafted to CNTs and graphene layers by transmission electron microscopy (TEM) allows the identification of the conformational carbon network arrangement towards common organic reactions used for the construction of molecular functional interfaces [14].

## 2. Experimental

### 2.1. Materials and reagents

All solvents and chemicals were purchased from Sigma-Aldrich and used without further purification. HiPCO single-walled carbon nanotubes (SWCNTs) were obtained from Carbon Nanotechnology, Inc. (lot no. R0496, www.cnanotech.com). Multi-walled carbon nanotubes (MWCNTs) were purchased from NANOCYL NC7000 (www.nanocyl.com). Graphite was purchased from Bay Carbon, Inc. (SP-1 graphite powder, batch no. 04100, lot no. 011705) www.baycarbon.com). Gold (III) chloride hydrate solution (99.99% trace metal basis, Aldrich) was used as a metal precursor (0.05 mM).

### 2.2. Characterization techniques

TEM images were acquired on a TEM JEOL JEM-2100, using an accelerating voltage of 200 kV. Samples were prepared by drop casting of stable dispersions onto a TEM grid (200 mesh, Lacey carbon films). Raman scattering was measured with a Thermo Scientific DXR Raman microscope equipped with a diode-pumped solid-state laser at a wavelength of 532 nm as an excitation source. It had a 20× objective with a 50 µM slit aperture, 7 s of exposure time and laser power of 10 mW. Samples were prepared by drop casting of the dispersion on silicon oxide surfaces (Si-Mat silicon wafers, CZ) and the solvent was allowed to evaporate. For Raman analysis 30 spectra were taken of each sample. The chemical composition of the samples was analysed by X-ray photoelectron spectroscopy (XPS) using an XPSVERSAPROBE PHI 5000 from Physical Electronics, equipped with a monochromatic Al Kα X-ray source under UHV conditions. The energy resolution was 0.7 eV. For the compensation of built-up charge on the sample surface during the measurements, a dual beam charge neutralization composed of an electron gun (**B**1 eV) and an argon ion gun (**r**10 eV) was used. The XPS spectra were deconvoluted into different chemical surroundings using commercially available software (CASA-XPS).

### 2.3. Synthesis of AuNPs

400 µM HAuCl_4_ in deionized water–methanol solution (1M, 80 ml) was irradiated with UV light (GE. R 500 W Helios Italquartz, UVB, UVA ozone free emission 310–450 nm, *λ*_max_ = 360 nm) for 60 min under magnetic stirring. The products were washed by centrifugation to remove all the by-products of the reaction. The average size distribution is 36 ± 9 nm [15].

### 2.4. Purified CNTs (p-SWCNTs and p-MWCNTs)

300 mg of pristine CNTs was sonicated for 5 min in 300 ml of 2.6 M solution of nitric acid and refluxed for 48 h at 125°C under magnetic stirring. The solution was neutralized, filtered (Millipore JH 0.45 µm filter) and washed with deionized water. p-CNTs were washed with methanol and dried in vacuum overnight (249 mg, 83%).

### 2.5. CNTs oxidation (ox-SWCNTs and ox-MWCNTs)

After purification, p-CNTs devoid of metallic catalytic particles were sonicated in piranha solution for 30 min and washed thoroughly until reaching pH = 4.5 [16].

### 2.6. Amine functionalized CNTs (f-SWCNTs and f-MWCNTs)

Boc-protected derivatives were prepared as follows: p-CNTs (120 mg) were dispersed and homogenized in distilled water (100 ml) by sonication. Then 4-[(N-Boc) aminomethyl] aniline (2 g, 8.9 mmol) and isoamyl nitrite (2 ml, 14.8 mmol) were added to the suspension and the reaction was refluxed at 80°C overnight. The suspension was filtered and then washed with *N*,*N*-dimethylformamide (DMF) and methanol until the solutions were clear of any impurity. The obtained black solid was dried under vacuum overnight (170.1 mg). The cleavage of the Boc groups was carried out by their dispersion in 4 M hydrochloric acid (HCl) using 1,4-dioxane as the solvent. The reaction was kept stirred at room temperature overnight and then it was filtered and washed thoroughly with DMF and methanol. Then the completely cleaned samples were dried (140.1 mg). The presence of amino terminal groups was verified by using the Kaiser test before and after the de-protection of the Boc group, obtaining an average of 124 ± 54 and 297 ± 75 µmol g^−1^ of f-SWCNTs and f-MWCNTs, respectively [17].

### 2.7. Photodeposition of AuNPs on CNT (AuNP@p-SWCNTs, AuNP@p-MWCNTs, AuNP@ox-SWCNTs and AuNP@ox-MWCNTs)

10 mg of the p-CNTs or ox-CNTs were sonicated in 10 ml of 1 M solution of methanol in water for 10 min. 1.5 M citric acid solution in water was used to adjust the pH to 1. HAuCl_4_ was used as metal precursors (0.05 mM, 400 µl). Water dispersions were deoxygenated with Ar to avoid photo-oxidation of the tubes. Solutions were irradiated with UV light for intervals of 60 min under magnetic stirring [13].

### 2.8. Exfoliation of graphite

Few-layer graphene (FLG) was produced by ultrasonication, using an ultrasonic tip processor GEX 750. Samples were sonicated in cycles of 30/30 s on/off for periods of 3 h, at the lower power of the ultrasonic tip (20%, 150 W). During ultrasonication, samples were kept in an ice bath to avoid overheating. After sonication, the FLG was washed and dispersed in DMF [18].

### 2.9. Graphene oxide synthesis

GO was obtained by oxidizing graphite crystals using the improved Hummers’ method reported by Marcano *et al*. [19]. A concentrated mixture of H_2_SO_4_ (360 ml) and H_3_PO_4_ (40 ml) was added to a mixture of 3 g of graphite and 18 g of KMnO_4_. The mixture was stirred for 12 h at 50°C. After this, the mixture was filtered using a 0.2 µm polytetrafluoroethylene (PTFE) membrane filter. The filtrate was centrifuged for 5 h at 4000 r.p.m. and the supernatant was discarded; the resultant material was then washed with 200 ml of deionized water, 200 ml of 30% HCl and 200 ml of ethanol, and it was filtered using a PTFE membrane filter. This washing process was repeated three times. The remaining material was dispersed in 200 ml of ether, and the resulting suspension was filtered again with a PTFE membrane filter. The solid obtained on the filter was finally dried in vacuum for 24 h.

### 2.10. Amine functionalized graphene

Boc derivative was prepared as follows: 5 ml of water was added to 25 ml of colloidal graphene dispersion (DMF/H_2_O, 5 : 1); the reagents 4-[(N-Boc) aminomethyl] aniline (2 equiv. per FLG) and isoamyl nitrite (5 equiv. per FLG) were added to the suspension and the reaction was refluxed at 80°C overnight. The resulting solution was cooled down to room temperature, filtered (PTFE membrane, 0.2 µm), washed thoroughly and dispersed for the cleavage of the Boc groups in 4 M hydrochloric acid (HCl) using 1,4-dioxane as the solvent. The reaction was kept stirred at room temperature overnight and after that it was filtered and washed thoroughly with DMF. The presence of amino terminal groups was verified by using the Kaiser test before and after the de-protection of the Boc group, obtaining an average of 653 ± 65 µmol g^−1^ of free amino terminal groups [20].

### 2.11. Photodeposition of AuNPs on GO (AuNP@GO)

Two milligram of GO was dispersed in 80 ml of a 1 M solution of methanol in water. A citric acid solution (1.5 M) was used to adjust the pH to 4.5. AuHCl_4_ was used as a metal precursor (0.05 mM, 400 µl). The solution was irradiated with UV light for 60 min under magnetic stirring.

### 2.12. AuNP recognition (AuNP@f-SWCNTs, AuNP@f-MWCNTs, and AuNP@f-FLG)

One millilitre of f-CNTs and f-FLG dispersions in fresh DMF were mixed with 1 ml of AuNPs. Dispersions were allowed to stabilize under magnetic stirring overnight [21].

## 3. Results and discussion

**Figure 1.**
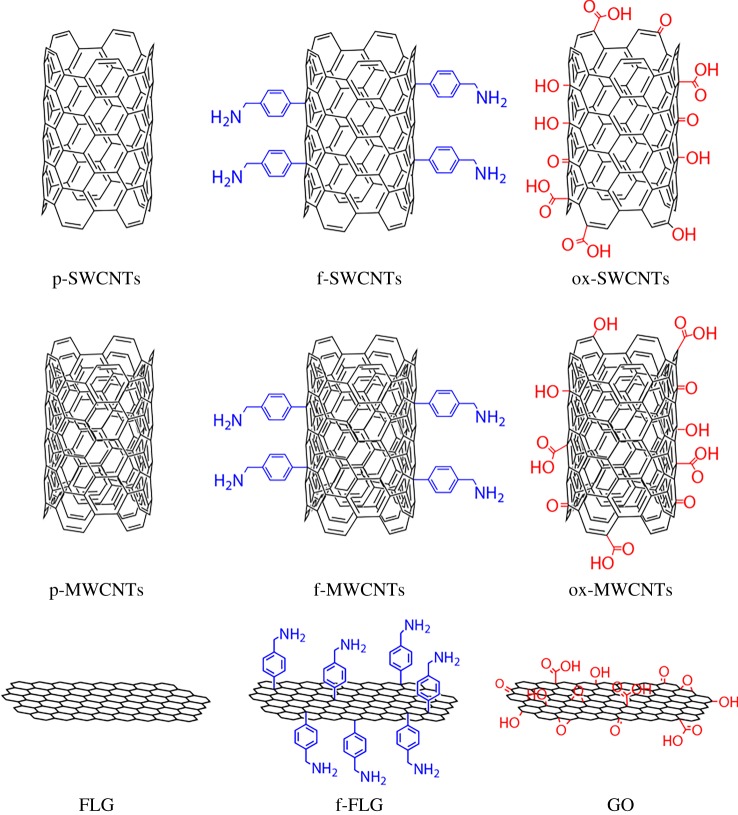
Schematic of carbon nanostrutures.

The lack of solubility of most carbon nanostructures renders difficult their manipulation, imposing limitations to their use. The introduction of functional moieties such as amino, carboxylic or thiol groups on the surface of carbon nanostructures has aided to solve this problem [22,23]. Amino and carboxylic groups can be positively or negatively charged, introducing hydrophilic characteristics. In addition, a relatively easy coupling chemistry can be applied on these terminal functions, linking nearly any desired functionality [24]. In a similar approach, functional small molecules covalently attached to carbon nanostructures are used to stabilize AuNPs [21]. In this work, terminal amino or carboxylic functional groups are introduced on CNTs and graphene by applying two chemical reactions: a diazonium-based arylation reaction and oxidation in strong acids, respectively. Pristine and functionalized systems were then conjugated with AuNPs in colloidal dispersions and characterized by Raman and XPS spectroscopies before the visualization of the samples by TEM. The interaction of AuNPs with purified nanomaterials (p-SWCNTs, p-MWCNTs and FLG) are compared with amino functionalized nanostructures (f-SWCNTs, f-MWCNTs and f-FLG) and oxidized ones (ox-SWCNTs, ox-MWCNTs and GO). A schematic representation of the functionalized carbon nanostructures is shown in figure 1. Purified and functionalized carbon nanostructures were carefully characterized by Raman and XPS spectroscopies.

Raman spectroscopy is used as a powerful tool in the structural characterization of carbon nanomaterials. The strongest Raman band of graphitic materials is the G-band (tangential mode at around 1600 cm^−1^); weaker bands are the D-band (disorder or defect-induced band at around 1350 cm^−1^) and the 2D band at around 2600–2800 cm^−1^. The intensity of the D-band relative to the G-band is often used as a measure of the quality of the carbon nanostructure and as an indicator of chemical functionalization. The higher the D-band intensity, the stronger is the functionalization. In pure graphene the D-band originates from a hybridized vibrational mode associated with the edges, defects and adatoms [25].

The chemical composition of the samples was analysed by XPS. The overlap between C–O and C–N features makes difficult the clear identification of the amine funtionalites covalently grafted to the carbon networks. For this reason, the amount of free NH_2_ groups in functionalized nanostructures is identified by the Kaiser test.

**Figure 2.**
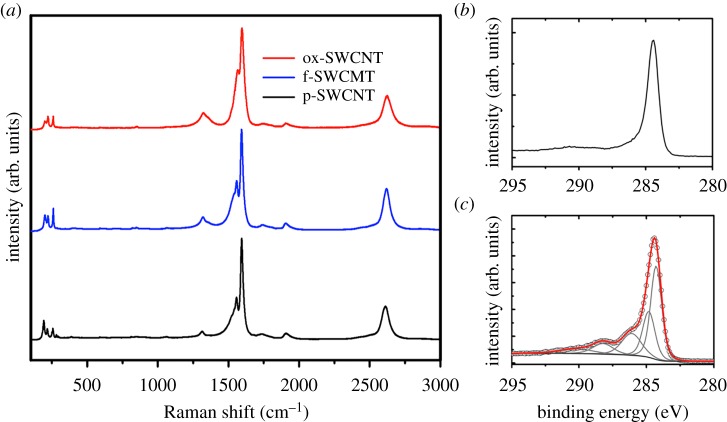
(*a*) Raman spectroscopy of p-SWCNTs, f-SWCNTs and ox-SWCNTs. (*b*) C 1s core-level photoemission line for p-SWCNTs. (*c*) C 1s core-level photoemission line for ox-SWCNTs.

In figure 2*a*, the Raman spectra of p-SWCNTs, f-SWCNTs and ox-SWCNTs are displayed. The two most prominent peaks are at 1584 and 2700 cm^−1^. These signals are attributed to the G and 2D bands, respectively. The radial breathing modes (RBMs) for SWCNTs appear around 250 cm^−1^. The signal at 1350 cm^−1^ is associated to the D band. For p-SWCNTs the signal corresponding to the D band has a low intensity, which indicates a very low amount of defects, while f-SWCNTs and ox-SWCNTs display broader and higher D band intensities; the *I*_D_/*I*_G_ ratios are 0.01, 0.14 and 0.16, respectively. In f-SWCNTs an aryl diazonium-based reaction in water introduces amino terminal groups by the transformation of a diazonium group into a radical capable of grafting covalently to the CNTs, while oxidation in ox-SWCNTs attacks the surface of the tubes, breaking the graphitic lattice and introducing carboxylic, ketone, alcohol and aldehyde functionalities among others. The narrowed 2D band of f-SWCNTs accounts for better dispersibility of the tubes compared with p-SWCNTs and ox-SWCNTs.

In purified samples, shown in figure 2*b*, the C 1s spectrum presents a main feature at 284.3 eV and a secondary feature corresponding to a p plasmon excitation centred at 291 eV. The shoulder at the high-energy side of the C 1s peak for ox-SWCNTs (shown in figure 2*c*) is generated by photoelectrons emitted from C atoms in oxygenated groups: hydroxyl (component centred at 286.2 eV), carbonyl (287.2 eV) and carboxyl groups (288.9 eV). The chemical composition of p-SWNCTs is C (99.3%) and O (0.7%). For f-SWCNTs, the Kaiser test value of 125 µmol g^−1^ of free NH_2_ in combination with the XPS of p-SWCNTs corresponds to a chemical composition of C (99%), O (0.7%) and N (0.15%), while ox-SWCNTs contains C (84%) and O (16%). As we have previously reported [13], AuNP@ox-SWNCT shows a reduction in the intensity of the C 1 s satellite peaks due to oxygenated groups. This signal reduction demonstrates that AuNPs are selectively grown on the oxygen-rich areas forming C-O**-**Au bonds.

**Figure 3.**
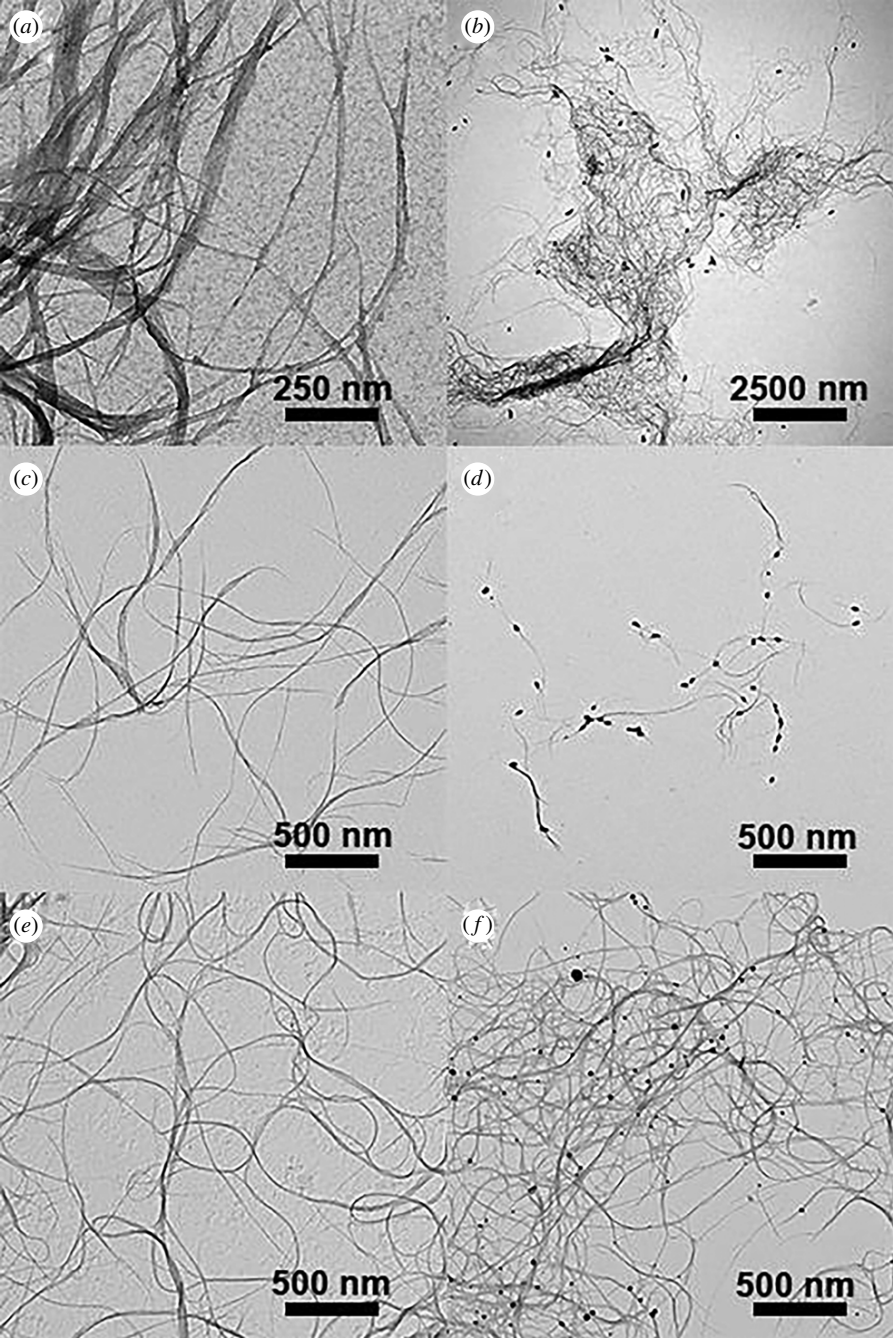
TEM micrographs of (*a*) p-SWCNT, (*b*) AuNP@p-SWCNT, (*c*) f-SWCNT, (*d*) AuNP@f-SWCNT, (*e*) ox-SWCNTs and (*f*) AuNP@ox-SWNCTs.

TEM micrographs of p-SWCNTs, AuNP@p-SWCNTs, f-SWCNTs, AuNP@f-SWCNTs, ox-SWCNTs and AuNP@ox-SWCNTs are shown in figure 3. Figure 3*a* shows aggregates of p-SWCNTs formed by π–π stacking interactions. In figure 3*c*, f-SWCNTs are dispersed in smaller bundles than those formed by ox-SWCNTs as shown in 3e. In figure 3*b*, a small number of AuNPs can be observed on the p-SWCNTs. At closer inspection, AuNPs appear mostly at the tips, which are in principle regions of defect abundance (oxygenated groups) [16]. In addition, AuNPs are observed dispersed on the TEM grid. The lack of defects or functional groups in the surface of the p-SWCNTs results in a very low amount of AuNPs on the sample. Figure 3*c* shows a higher density of AuNPs distributed on the f-SWCNTs’ small bundles. In figure 3*f*, a similar quantity of AuNPs is observed for ox-SWCNTs, although the aggregation is larger in the last one. These results are consistent with the Raman I_D_/I_G_ analysis. The formation of smaller bundles for f-SWCNTs compared to ox-SWCNTs might result from the fact that oxidation mainly occurs close to defects, amorphous carbon or on the tips of SWCNTs, while the organic arylation reaction attacks the aromatic carbon network [26].

**Figure 4.**
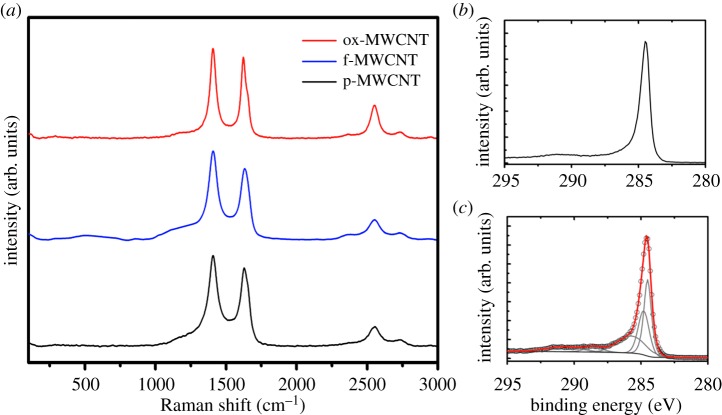
(*a*) Raman spectroscopy of p-MWCNTs, f-MWCNTs and ox-MWCNTs. (*b*) C 1s core-level photoemission line for p-MWCNTs. (*c*) C 1s core-level photoemission line for ox-MWCNTs.

Figure 4 shows the Raman spectra of p-MWCNTs, f-MWCNTs and ox-MWCNTs. The Raman spectroscopy of MWCNTs is totally different from that of SWCNTs; the RBM region is completely absent because nested tubes of different ratios are analysed. This fact resulted in the D band at 1350 cm^−1^ as the most prominent signal for MWCNTs making the Raman analysis difficult; the *I*_D_/*I*_G_ ratios are 1.15, 1.26 and 1.13 for p-MWCNTs, f-MWCNTs and ox-MWCNTs, respectively.

The XPS analysis gives similar information as the one reported for SWCNTs. Additional bands compared with the purified material were obtained for ox-MWCNTs. These bands correspond to hydroxyl (286.2 eV), carbonyl (287.2 eV) and carboxyl groups (288.9 eV). The composition of p-MWCNTs is C (99.4%) and O (0.6%). The Kaiser test value of 297 µmol g^−1^ for f-MWCNTs combined with the XPS of p-MWCNTs relate to a composition of C (99.05%), O (0.6%) and N (0.35%). After the oxidation treatment, the chemical composition of ox-MWCNTs changes to C (90.8%) and O (9.2%).

**Figure 5.**
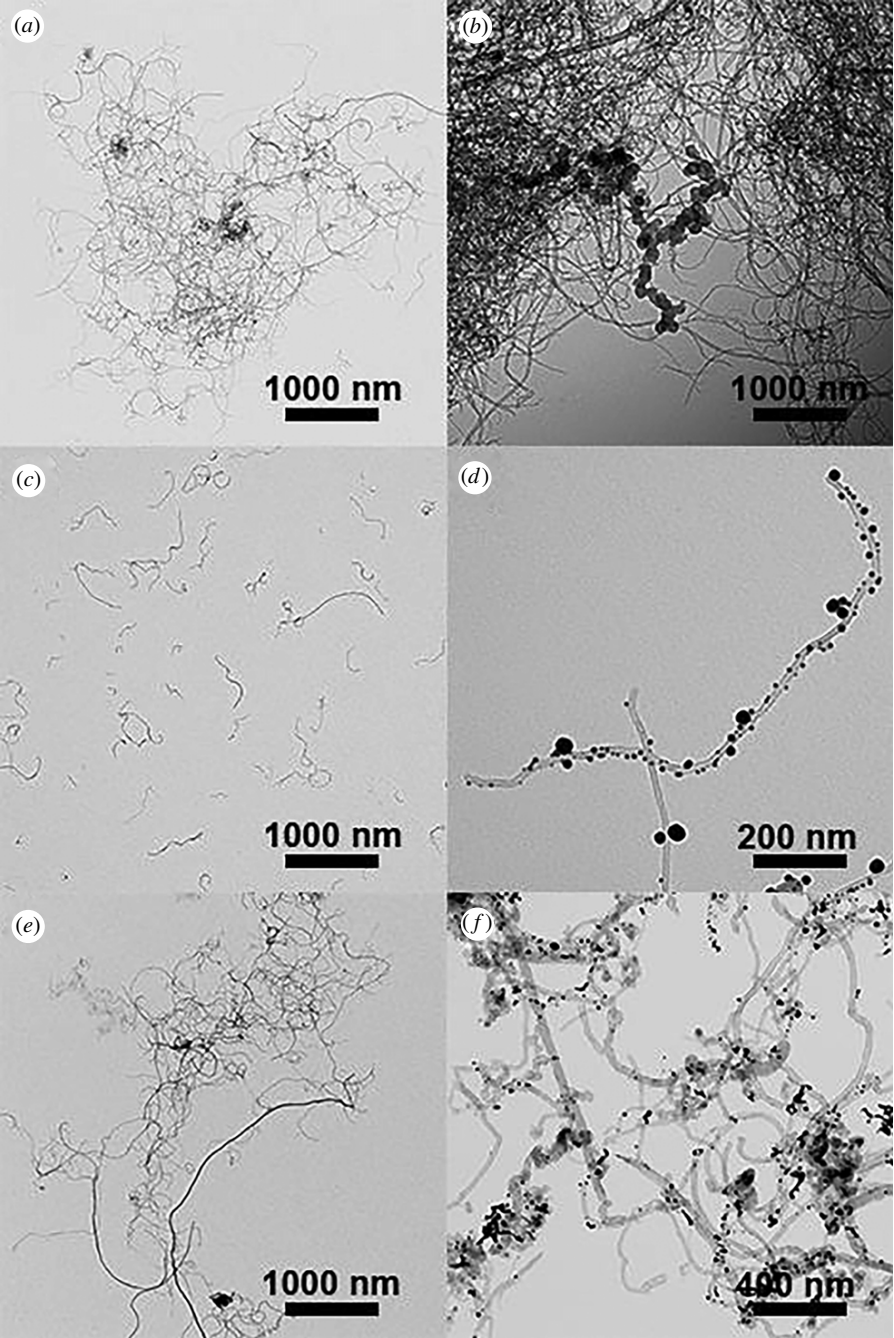
TEM micrographs of (*a*) p-MWCNTs, (*b*) AuNP@p-MWCNT, (*c*) f-MWCNTs, (*d*) AuNP@f-MWCNTs, (*e*) ox-MWCNT and (*f*) AuNP@ox-MWCNT.

TEM micrographs of p-MWCNTs, AuNP@p-MWCNTs, f-MWCNTs, AuNP@f-MWCNTs, ox-MWCNTs and AuNP@ox-MWCNTs are reported in figure 5. Figure 5*a* shows large aggregates of p-MWCNTs; the growth of AuNP on these samples resulted in spherical AuNPs completely separated from p-MWCNTs, as observed in figure 5*b*. The lack of defects on the p-MWCNTs prevents the nucleation of the AuNPs on their surface. Instead, f-MWCNTs produced completely dispersed individual tubes; as observed in figure 5*c*, the terminal amino functional groups induced the whole stabilization of the tubes in DMF, allowing the further recognition of AuNPs. In f-MWCNTs, higher degree of funtionalization is produced compared with f-SWCNTs; the results are confirmed by the Kaiser test. The higher functionalization is responsible for the higher dispersibility and the higher amount of AuNPs deposited on the surface of f-MWCNTs. The strong chemical treatment in ox-MWCNTs caused the oxidation of impurities in the sample and the addition of functional groups on the surface of the CNTs, like ketone, carboxyl and hydroxyl groups. This procedure reduces the hydrophobicity of the CNTs, enabling their dispersion in aqueous media. However, as observed in figure 5*e,* ox-MWCNTs are less dispersed than f-MWCNTs; the most probable explanation for this observation is again that oxidation reactions mostly occur on the amorphous carbon residues, close to defects on the carbon skeleton and on the tips of the tubes with higher curvature. The ox-MWCNTs show higher concentration of AuNPs distributed on the tip of the tubes and in the zones where amorphous carbon is still present, as seen in figure 5*f*. More images are reported in the electronic supplementary material. Although f-MWCNTs exhibit a lower functionalization degree than ox-MWCNTs, the first appear as well-dispersed tubes (figure 5*c*) completely covered by AuNPs (figure 5*d*), while the oxidized ones remain as smaller bundles, confirming that oxidation occurs at the residual amorphous carbon (figure 5*f*) mostly close to the defects. The same trend was observed for SWCNTs.

**Figure 6.**
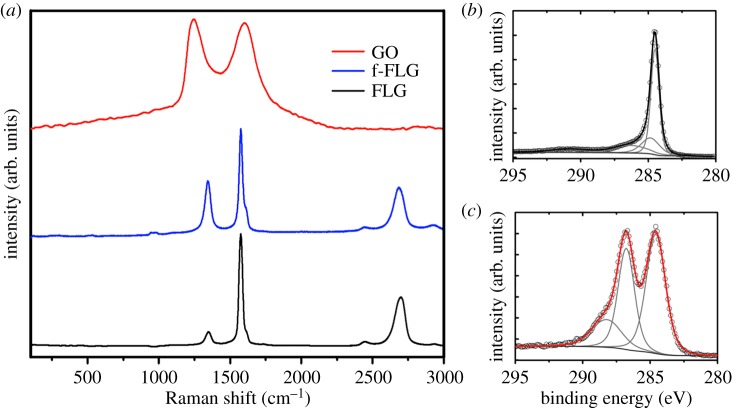
(*a*) Raman spectroscopy of FLG, f-FLG and GO. (*b*) C 1s core-level photoemission line for FLG. (*c*) C 1s core-level photoemission line for GO.

In figure 6*a*, the Raman spectra of FLG, f-FLG and GO are diplayed. A clear difference between the spectra is observed. Two characteristic peaks are located approximately at 1350 and 1590 cm^−1^; these peaks are attributed to the D and G band, respectively. In the Raman spectra, it is clearly observed that the peak associated with the D band considerably increases its intensity from FLG to f-FLG and finally to GO, indicating a higher concentration of functional groups. The I_D_/I_G_ ratios are 0.12, 0.53 and 1.04 for FLG, f-FLG and GO, respectively. The 2D band is located at approximately 2700 cm^−1^. The position and shape of this band are highly sensitive to the number and thickness of the graphene layers [20]. The signal associated with the 2D band obtained for FLG and f-FLG exhibited well-dispersed sheets. In GO, the 2D band is completely absent as a result of the strong degree of functionalization consistent with the broad and intense D band.

The XPS characterization for FLG and GO are reported in figure 6. In FLG (figure 6*b*), the main peak at 284.8 eV is attributed to the binding energy of photoelectrons emitted from sp^2^ C atoms. A secondary peak appears at 286.2 eV related to C–O and C–N bonds. The chemical composition is C (96.7%), O (2.5%) and N (0.7%). The presence of N in the sample derives from the non-covalent adsorption of the solvent (DMF) on FLG produced by the sonication process [20]. In GO, the presence of oxidized groups increased considerably, with three components corresponding to the following: (1) C–O and C–N are revealed at 286.2 eV, (2) C=O bonds shown by the peak at 287 eV and (3) O–C=O species at 288.0 eV. As expected, the GO spectrum shows two main peaks due to the high amount of oxygenated functionalities. In f-FLG, the chemical composition obtained by the combination of the Kaiser test value and XPS analysis of FLG is C (95.8%), O (2.5%) and N (0.8%). The percentage of N corresponded only to the covalently attached free NH_2_ functionalities because the Kaiser test blank was FLG. The chemical composition is C (69.3%) and O (29.3%).

**Figure 7.**
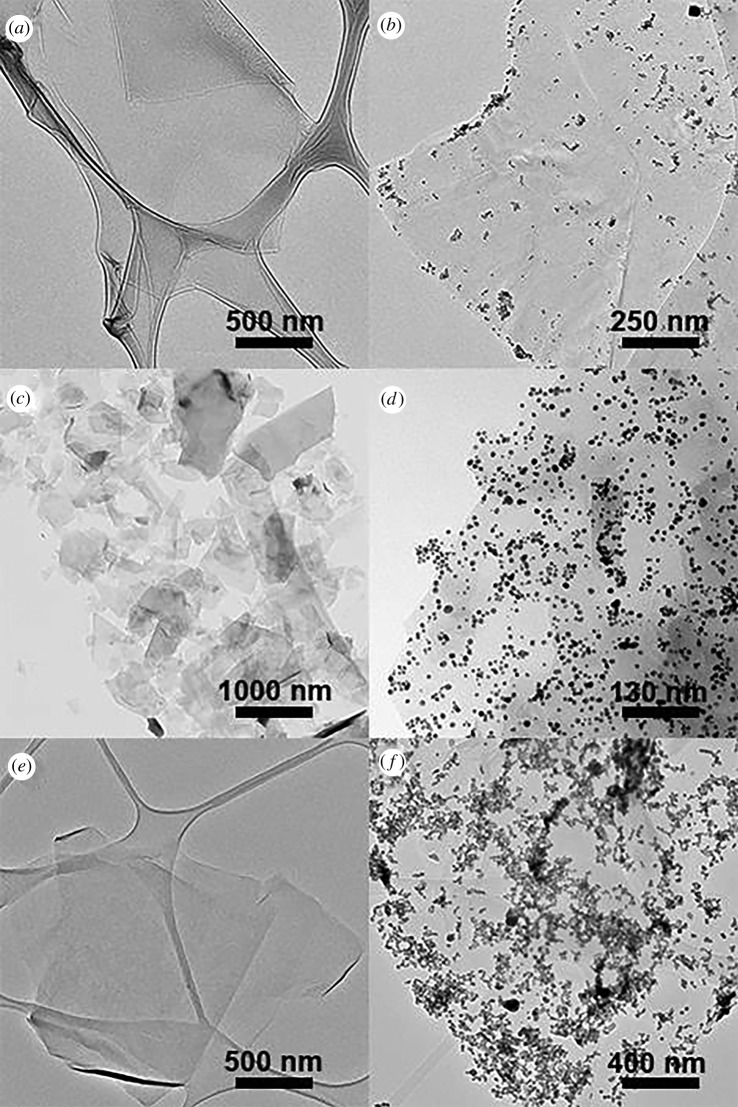
TEM micrographs of (*a*) FLG, (*b*) AuNP@FLG, (*c*) f-FLG, (*d*) AuNP@FLG, (*e*) GO and (*f*) AuNP@GO.

TEM analysis clearly corroborates the Raman spectroscopy. In figure 7, TEM images of FLG, AuNP@FLG, f-FLG, AuNP@FLG, GO and AuNP@GO are displayed. FLG relatively free of defects is observed in figure 7*a*. The UV-light irradiation produced the growth of AuNPs mainly at the edges of FLG, as shown in figure 7. At a closer view, the borders of smaller and superficial sheets are responsible for the observation of AuNPs on the inner part of GO. The functionalization with amino terminal groups of f-FLG induces higher dispersibility of smaller sheets; figure 7*c*. As observed in figure 7*d,* AuNPs are completely dispersed on f-FLG. Finally, the higher oxidation of GO produced the growth of AuNPs on the whole sheets in the form of aggregated NPs, denoting higher and localized chemical functionalization. More images are reported in the electronic supplementary material. These results are in complete agreement with Raman and XPS analysis. TEM images confirm that defects and oxygenated functions in FLG are mostly distributed at the edges. As for SWCNTs and MWCNTs, pointed covalent chemical functionalization results in materials with less amount of attached molecules randomly distributed on the complete surface. The homogeneous distribution of the functionalities allows to obtain well-dispersed f-FLG in organic solvents. However, aggressive oxidation reactions produce highly oxidized clusters of carbon atoms, resulting in lower dispersibility of GO compared with f-FLG. Importantly, FLG is a more chemically reactive surface compared to SWCNTs and MWCNTs, most probably as a consequence of concerted cooperative reactions occurring on the 2D surface [27].

## 4. Conclusion

In this work, two well-characterized covalent organic reactions, namely diazonium-based arylation reaction and chemical oxidation in solution were successfully performed on SWCNTs, MWCNTs and FLG. The Kaiser test, and Raman and XPS spectroscopies were used to corroborate the degree of functionalization and chemical composition achieved for each reaction on each type of nanocarbon. We have demonstrated that the conjugation of the systems with AuNPs allows the localization of the functional groups on the carbon frame, giving information of the structural configuration of the material. Pristine materials show the lowest density of AuNPs, consistent with the low amount of defects and functional groups mostly concentrated at the tips and edges. Functionalization of carbon nanostructures by a diazonium-based arylation reaction, however, introduces amino terminal groups randomly distributed on the whole surface of f-SWCNTs, f-MWCNTs and f-FLG, yielding higher dispersibility in polar organic solvents. Finally, in ox-SWCNTs, ox-MWCNTs and GO, the strong oxidation reaction generates clusters of oxygenated moieties close to defects, amorphous carbon, tips and edges, resulting in the growth of AuNPs at localized zones. In oxidized materials, the increase in the amount of AuNPs on carbon nanostructures is related with the higher concentration of oxygenated moieties as nucleation points for AuNP growth. The diazonium-based arylation reaction produced nanostructures presenting AuNPs well dispersed on the whole carbon network, while oxidation reactions caused the aggregation of AuNPs mostly close to the tips and edges of the carbon lattices where a higher concentration of defects is expected.

In summary, the final concentration and dispersion of AuNPs on carbon nanostructures is only dependent on the number of functionalities present in the carbon lattice, but not on the type of carbon nanostructure. We have shown that TEM characterization of carbon nanostructures conjugated with AuNPs is an important complementary tool for a better interpretation of the chemical structure of functionalized carbon nanomaterials. This strategy allows the direct observation and quantification of AuNPs attached to chemically oxygenated and aminated moieties. It is expected that this intepretation allows the tailored synthesis of novel functionalized carbon nanostructures for advanced applications in the biomedicine and energy fields.

## Supplementary Material

ESM 1 - 5

## Data accessibility

Additional TEM images of AuNP@CNS supporting this investigation have been uploaded as part of the electronic supporting information.
