# Supplementary material for: Imaging carbon nanostructures’ reactivity: a complementary strategy to define chemical structure
Source: R Soc Open Sci. 2018 Aug 8;5(8):180605. doi: 10.1098/rsos.180605 (PMC6124117; doi:10.1098/rsos.180605)

Imaging Carbon Nanostructures Reactivity: A Complementary Strategy to Define Chemical Structure

Verónica Pérez-Luna, Mario Cisneros, Carla Bittencourt, Izcoatl Saucedo-Orozco, and Mildred Quintana

Electronic Supplementary Material (ESM)

Single-Walled Carbon Nanotubes

1) p-SWCNTs

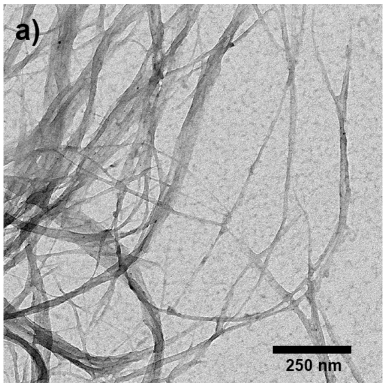

2) AuNP@p-SWCNTs

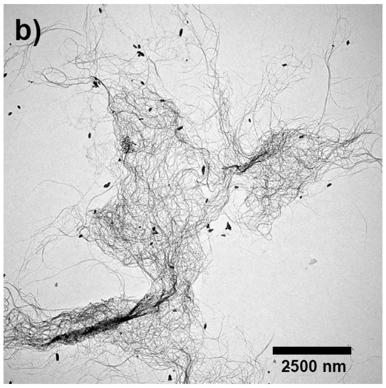

3) f-SWCNTs

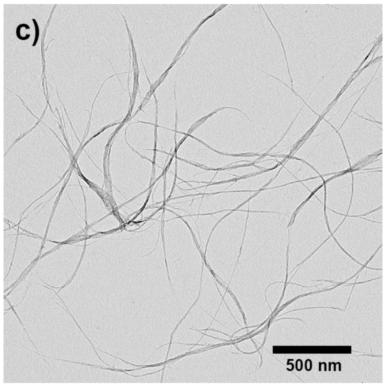

4) AuNP@f-SWCNTs

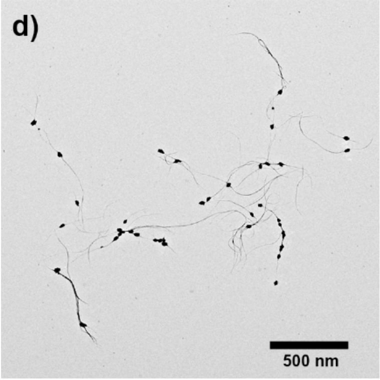

5) ox-SWCNTs

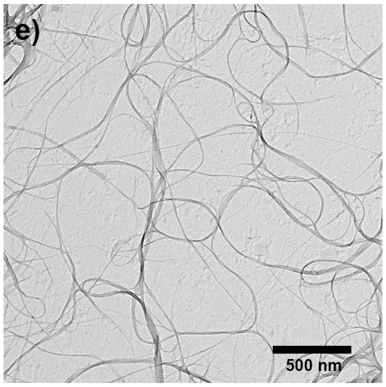

6) AuNP@ox-SWCNTs

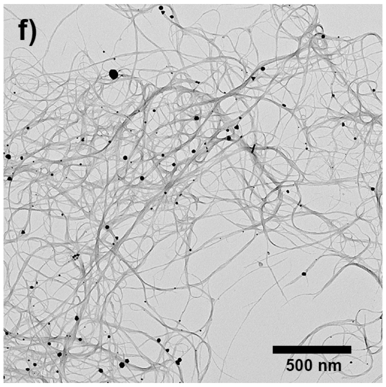

Multi-Walled Carbon Nanotubes

1) p-MWCNTs

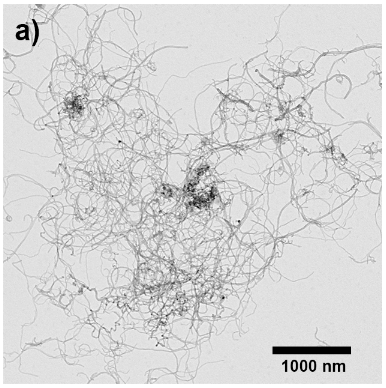

2) AuNP@p-MWCNTs

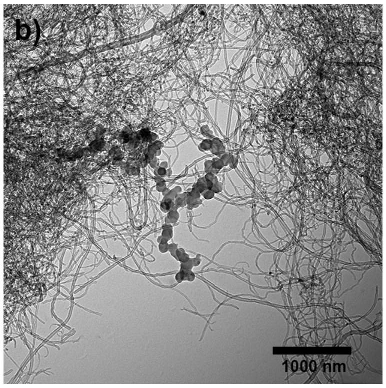

3) f-MWCNTs

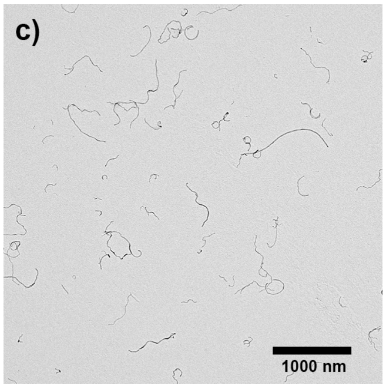

4) AuNP@f-MWCNTs

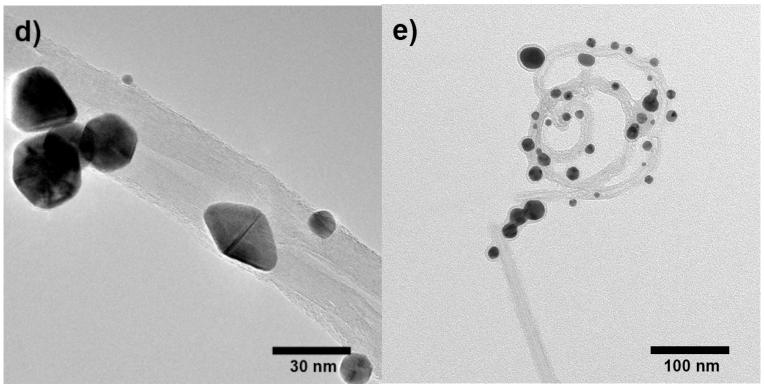

5) ox-MWCNT

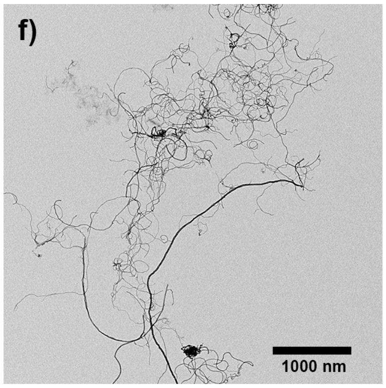

6) AuNP@ox-MWCNTs

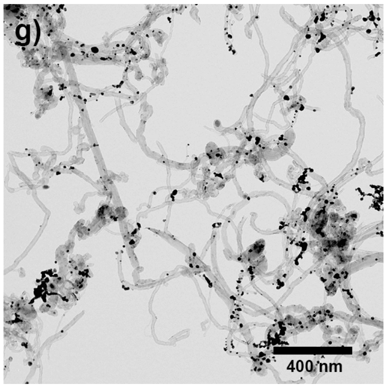

Grafeno exfoliado

1) FLG

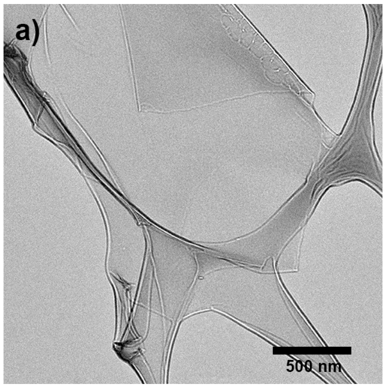

2) AuNP@FLG

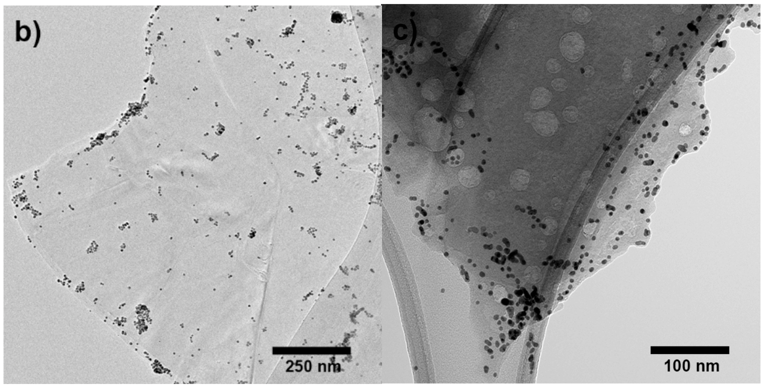

3) f-FLG

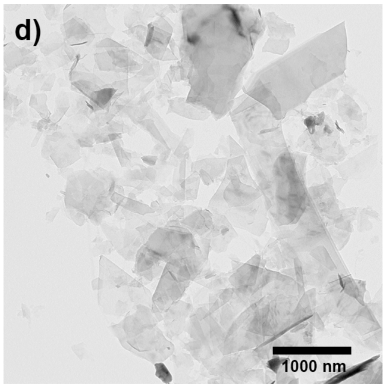

4) AuNP@f-FLG

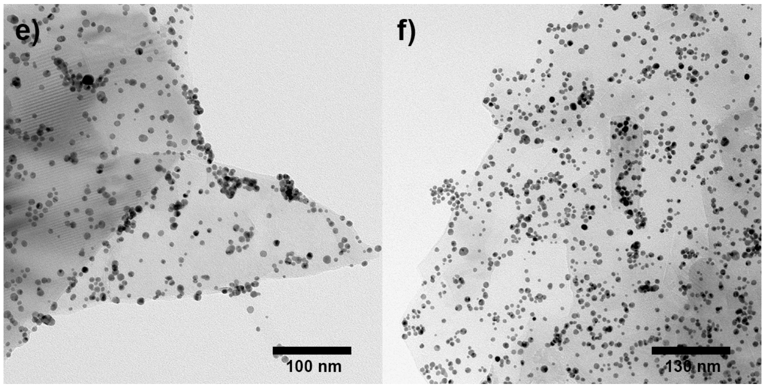

5) GO

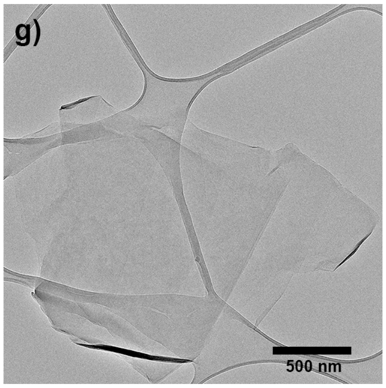

6) AuNP@GO

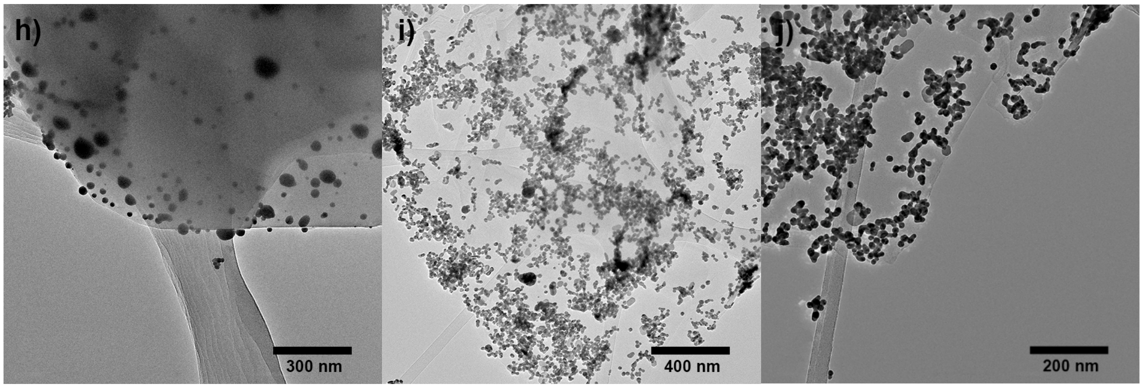

Supplement: ESM 1 - 5 [file rsos180605supp1.pdf]
